# Impact of rituximab on patient-reported outcomes in patients with rheumatoid arthritis from the US Corrona Registry

**DOI:** 10.1007/s10067-017-3742-2

**Published:** 2017-07-17

**Authors:** Leslie R. Harrold, Ani John, Jennie Best, Steve Zlotnick, Chitra Karki, YouFu Li, Jeffrey D. Greenberg, Joel M. Kremer

**Affiliations:** 1 University of Massachusetts Medical School, Worcester, MA USA; 2 Corrona, LLC, Southborough, MA USA; 3 Genentech, Inc., South San Francisco, CA USA; 4 New York University School of Medicine, New York, NY USA; 5 Albany Medical College and the Center for Rheumatology, Albany, NY USA

**Keywords:** Biologics, Patient-reported outcomes, Rheumatoid arthritis, Rituximab

## Abstract

To evaluate the impact of rituximab on patient-reported outcomes (PROs) in a US-based observational cohort of patients with rheumatoid arthritis (RA). Patients with active RA, prior exposure to ≥1 tumor necrosis factor inhibitor (TNFi) and who newly initiated rituximab were identified. Changes in PROs were assessed 1 year after rituximab initiation. PRO measures included Clinical Disease Activity Index (CDAI); patient global disease activity, pain and fatigue (visual analog score; 0–100); morning stiffness (hours); modified Health Assessment Questionnaire (mHAQ; 0–3); and EuroQoL EQ-5D. Of the 667 patients who newly initiated rituximab, baseline PRO and clinical measures indicated that patients were substantially impacted by their RA disease and quality of life; 54% of patients had high disease activity. One year after rituximab initiation, 49.0, 47.1, 49.8, and 23.2% of patients reported clinically meaningful improvements in patient global, pain, fatigue, and mHAQ, respectively. Morning stiffness and EuroQol EQ-5D domains improved in 48 and 19–32% of patients, respectively. These real-world registry data demonstrated that patients with long-standing, refractory RA experienced improvements in PROs 1 year after initiating rituximab.

**Electronic supplementary material:**

The online version of this article (doi:10.1007/s10067-017-3742-2) contains supplementary material, which is available to authorized users.

## Introduction

Patients with rheumatoid arthritis (RA) often experience reduced health-related quality of life (HRQOL), including disability and RA-related comorbidities [1, 2]. The goals of treatment in RA are to achieve low disease activity (LDA) or remission and to improve HRQOL.

Patient-reported outcomes (PROs) include HRQOL indices, such as the ability to perform day-to-day tasks, emotional health, and the degree of pain and discomfort. PROs are increasingly being recognized as important measures in determining response to therapy in patients with RA [3–5].

Rituximab is a monoclonal antibody that targets and depletes CD20^+^ B cells and, in combination with methotrexate, is approved for the treatment of RA in patients who have had an inadequate response to ≥1 tumor necrosis factor inhibitor (TNFi). Limited data exist on the effect of rituximab on PROs in real-world clinical settings. This study examined the impact of rituximab on PROs in a US observational cohort of patients with RA.

## Methods

### Study setting

The Corrona RA Registry is an independent, prospective, observational cohort of patients with RA [6, 7]. Patients are recruited from 169 private and academic practice sites across 40 states in the USA, with 656 participating rheumatologists. As of June 20, 2016, data on 43,099 patients with RA have been collected. The protocol was approved by the New England institutional review board (IRB; #120160610) and the local IRBs of participating academic sites. Registration number: NCT01402661.

### Study analysis population

Adult patients with RA who initiated rituximab for the first time within Corrona from March 2006 to September 2015 were identified. Eligible patients had available PRO measurements at baseline (around the time of rituximab initiation) and 1 year (9–15 months) after rituximab initiation. All patients had previously received ≥1 TNFi and had low, moderate, or high disease activity based on Clinical Disease Activity Index (CDAI), defined as CDAI >2.8.

### Assessments and outcomes

CDAI was assessed at 1 year and evaluated by the median change from baseline and by the proportion of patients achieving LDA/remission (CDAI ≤10). PROs were assessed at 1 year and included the median change from baseline and the proportion of patients reporting minimum clinically important differences (MCIDs; defined as improvement of ≥10) in patient global assessment of disease, pain, and fatigue (0–100 on a visual analog scale) [8]; improvement in morning stiffness (duration in hours); proportion of patients achieving a clinically meaningful improvement in modified Health Assessment Questionnaire (mHAQ), defined as a decrease of >0.25 from baseline in the mHAQ score [9]; and improvement in the EuroQol EQ-5D overall health status index, which records patient-reported HRQOL across five domains (walking, self-care, usual activities, pain/discomfort, and anxiety/depression). EuroQol EQ-5D results are examined using a summary index (0–1) or by individual evaluation of each domain [10]. Improvement in EQ-5D domains was defined as the proportion of patients with any improvement or resolution of impairment among patients who reported impairment at baseline.

PROs and CDAI were evaluated for both the overall cohort and in patient populations stratified by number of prior TNFis (1 or ≥2 prior TNFis). For patients with ≥2 visits within the time frame for the 12-month visit, the visit closest to 12 months was used.

### Statistical analysis

Comparisons between the 1 and ≥2 prior TNFi groups were performed using *χ* ^2^, *t*, or nonparametric equality-of-medians tests, as appropriate. Responses for the outcome measures were available for >95% of patients during the time when the variables were part of the data collection process; patients with missing data were excluded from outcome analyses.

## Results

### Patient demographics and baseline PROs

A total of 667 patients were included (Supplemental Fig. 1); 284 (42.6%) had received 1 prior TNFi, and 383 (57.4%) had received ≥2 prior TNFis. Most patients were female (78.7%), and the overall median age was 59 (interquartile range [IQR], 50–66) years (Table 1). The median duration of RA was 13 (IQR, 7–21) years, and 53.8% of patients had high disease activity (CDAI >22) at baseline. Significantly higher proportions of patients with ≥2 prior TNFis had received ≥1 non-TNFi biologic (53.8 vs 39.1%) and were in high disease activity (CDAI >22; 58.2 vs 47.9%) than patients with 1 prior TNFi.

**Table 1:** Baseline patient demographics, clinical characteristics, disease activity, and PRO measures

	Total *N* = 667	1 Prior TNFi *n* = 284	≥2 Prior TNFis *n* = 383	*P* value^a^
Demographics and clinical characteristics				
Age, median (IQR), years	59 (50–66)	60 (52–69)	58 (49–65)	0.013
Female, *n* (%)	525 (78.7)	223 (78.5)	302 (78.9)	0.918
White, *n* (%)	582 (87.3)	241 (84.9)	341 (89)	0.110
Duration of RA, median (IQR), years [*n*]	13 (7–21) [664]	11 (6–22) [281]	14 (7–27) [383]	0.071
History of cardiovascular disease, *n* (%)	42 (6.3)	17 (6.0)	25 (6.5)	0.776
History of diabetes, *n* (%)	61 (9.1)	26 (9.2)	35 (9.1)	0.994
History of hyperlipidemia, *n*/*N* (%)	22/317 (6.9)	10/129 (7.8)	12/188 (6.4)	0.638
RF seropositive, *n*/*N* (%)	290/395 (73.4)	123/164 (75.0)	167/231 (72.3)	0.549
No. of prior nonbiologic DMARDs, median (IQR)	1 (0–2)	1 (0–2)	2 (1–3)	<0.001
No. of prior non-TNFi biologics, %				
0	350 (52.5)	173 (60.9)	177 (46.2)	0.001
1	228 (34.2)	82 (28.9)	146 (38.1)	
≥2	89 (13.3)	29 (10.2)	60 (15.7)	
CDAI score, *n* (%)				
Low (>2.8 and ≤10)	81 (12.1)	38 (13.4)	43 (11.2)	0.029
Moderate (>10 and ≤22)	227 (34.0)	110 (38.7)	117 (30.5)	
High (>22)	359 (53.8)	136 (47.9)	223 (58.2)	
HRQOL measures, median (IQR) [*n*]				
Patient global assessment (0–100)	40 (25–60) [667]	40 (24.5–55) [284]	40 (25–60) [383]	0.302
Patient pain (0–100)	60 (31–75) [667]	50 (25–75) [284]	60 (36–75) [383]	0.084
Patient fatigue (0–100)	65 (40–80) [295]	55 (26–75) [123]	70 (48.5–85) [172]	0.014
mHAQ score (0–3)	0.6 (0.3–1) [660]	0.6 (0.3–1) [283]	0.8 (0.3–1) [377]	0.094
Morning stiffness, hours	1 (0.5–2) [650]	1 (0.5–2) [277]	1.5 (0.5–2.8) [373]	0.019
EQ-5D (0–1)	0.7 (0.6–0.8) [274]	0.7 (0.6–0.8) [114]	0.7 (0.6–0.8) [160]	0.228

*CDAI* clinical disease activity index, *DMARD* disease-modifying antirheumatic drug, *HRQOL* health-related quality of life, *IQR* interquartile range, *mHAQ* modified Health Assessment Questionnaire, *PRO*, patient-reported outcome, *RA* rheumatoid arthritis, *RF* rheumatoid factor, *TNFi* tumor necrosis factor inhibitor

^a^The *P* value represents the comparison between patients who received 1 prior TNFi vs those who received ≥2 prior TNFis

Patients were substantially impacted by their RA at baseline. Overall, baseline median (IQR) scores for patient global assessment, pain, and fatigue were 40 (25–60), 60 (31–75), and 65 (40–80) mm, respectively (Table 1). The baseline median mHAQ score was 0.6 (IQR, 0.3–1), and patients reported a median of 1 (IQR, 0.5–2) hour of morning stiffness. Most patients reported at least some problems in the EQ-5D categories of walking (75.7%), usual activities (81.1%), and pain/discomfort (95.8%). Almost one-half of all patients reported at least some problems in self-care (48.6%) and anxiety/depression (49.0%). Baseline PRO scores were mostly similar between patients who had received 1 or ≥2 prior TNFis, with the exception of fatigue and morning stiffness.

### Rituximab persistency

Overall, 78.9% of patients persisted on rituximab through 1 year, whereas 21.1% switched to another biologic before 1 year. Among all patients, 63.3% received rituximab retreatment and did not switch to another biologic, and 15.6% did not receive rituximab retreatment but also did not switch to another biologic (Supplemental Table 1).

### Improvement in CDAI and PROs 1 year after rituximab initiation

The median improvement in CDAI from baseline to 1 year was 8 (IQR, 17.8); results were similar between patients with 1 or ≥2 prior TNFis. Overall, 30.4% of patients achieved CDAI LDA/remission (CDAI ≤10) at 1 year. A significantly higher proportion of patients with 1 prior TNFi achieved CDAI LDA/remission than patients with ≥2 prior TNFis (37.7 vs 25.1%, respectively; *P* < 0.001).

**Fig. 1:**
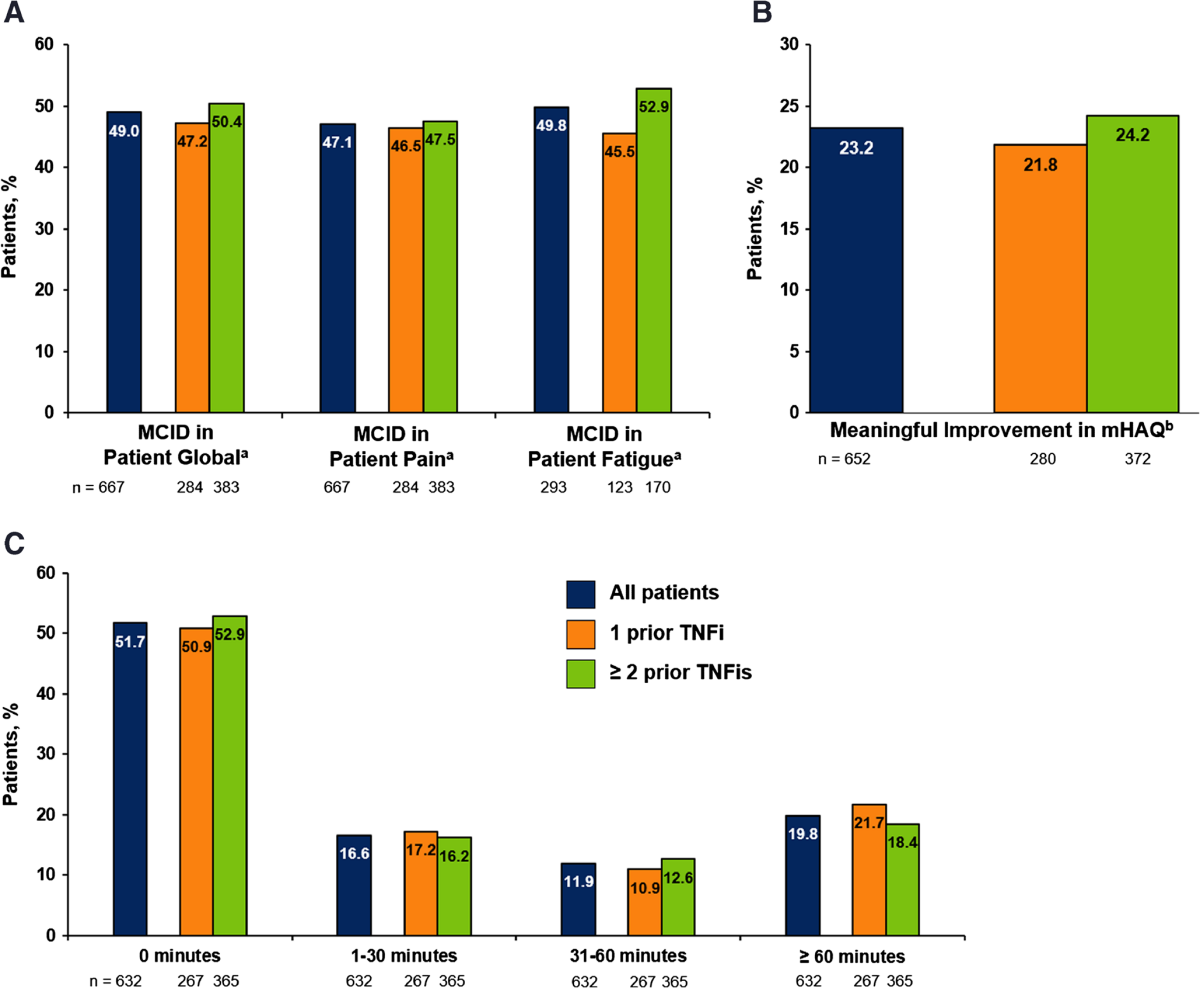
Improvement in PROs (**a**), mHAQ (**b**), and duration of morning stiffness (**c**)1 year after initiation of rituximab, overall and by prior TNFi use. *MCID* minimum clinically important difference, *mHAQ* modified Health Assessment Questionnaire, *PRO* patient-reported outcome, *TNFi* tumor necrosis factor inhibitor. *a* MCIDs for patient global assessment, pain, and fatigue were defined as median improvement from baseline to 1 year of ≥10 on a visual analog scale (0–100). *b* Meaningful improvement in mHAQ score was defined as an improvement of >0.25.

Improvement from baseline was observed in all PRO measures at 1 year. The overall median (IQR) scores of patient global assessment, pain, and fatigue improved by 7 (35), 7 (30), and 9 (25) mm, respectively. The proportions of patients with improvements ≥MCID in patient global assessment, pain, and fatigue were 49.0, 47.1, and 49.8%, respectively (Fig. 1a). Clinically meaningful improvement in mHAQ was reported in 23.2% of patients (Fig. 1b). Almost one-half of all patients (48.3%) reported some improvement in morning stiffness, with 19.8% reporting a reduction in the duration of morning stiffness of >60 min (Fig. 1c).

**Fig. 2:**
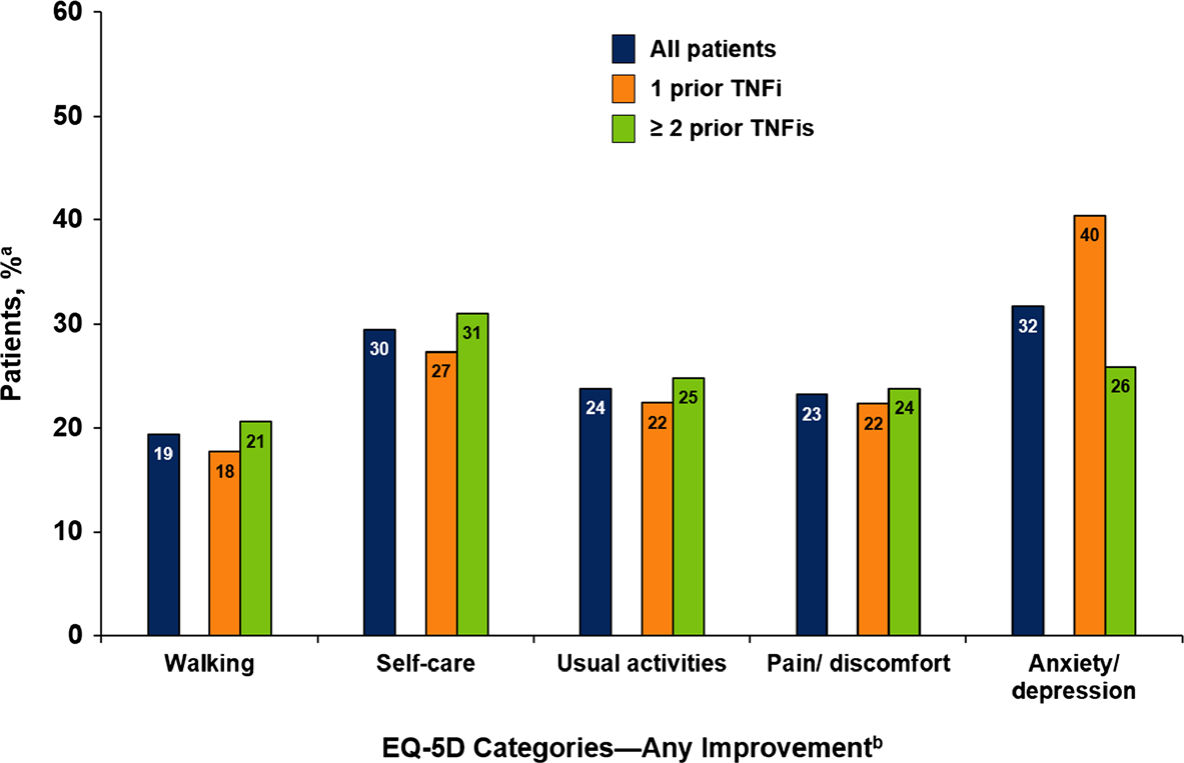
Improvement in EQ-5D categories at 1 year among rituximab initiators, overall and by prior TNFi use. *TNFi* tumor necrosis factor inhibitor. *a* Percentage of patients reporting improvement among patients who reported difficulty in each measure at baseline. *b* Improvement in the EQ-5D domains was defined as either patients improving from moderate to no disability or those with severe disability improving to moderate or no disability

Patients also experienced improvement in all EQ-5D domains at 1 year; among patients who reported problems at baseline, 19% of patients reported at least some improvement in walking, 30% in self-care, 24% in usual activities, 23% in pain/discomfort, and 32% in anxiety/depression (Fig. 2). Among patients who reported problems at baseline, 19% of patients reported no problems in walking, 27% in self-care, 18% in usual activities, 11% in pain/discomfort, and 32% in anxiety/depression. Similar improvements were observed between patients who received 1 prior TNFi and those who received ≥2 prior TNFis.

## Discussion

In this real-world cohort of patients with long-standing, refractory RA with active disease and prior TNFi exposure, improvements were reported in all PROs 1 year after initiation of rituximab. The overall median duration of RA was 13 years; whereas all patients had prior TNFi exposure, almost one-half (47.5%) had also received ≥2 prior non-TNFi biologics. At baseline, 88% of patients had moderate or high disease activity based on CDAI, and patients were substantially impacted by their RA: most patients reported problems with walking, usual activities, and pain/discomfort, and almost one-half reported problems with self-care and anxiety/depression. One year after initiation of rituximab, 30% of patients achieved LDA/remission (CDAI ≤10), and 49.0, 47.1, and 49.8% of patients achieved MCIDs in patient global, pain, and fatigue, respectively. Among patients who had reported problems at baseline, 11–32% reported no problems in walking, self-care, usual activities, pain/discomfort, and anxiety/depression at 1 year.

Clinical trials have also reported improvements in PROs among patients with comparable disease duration and prior use of TNFis; however, these studies reported different PRO measures, including HAQ—disability index, Functional Assessment of Chronic Illness Therapy—Fatigue, and Short Form 36, 6 months after initiation of rituximab, limiting the ability to directly compare our results with clinical trial results [11, 12]. Real-world data on PROs in rituximab-treated patients with RA are limited. A recent open-label study of rituximab in patients with long-standing RA showed that improvement in PROs occurred early after initiation of rituximab, plateaued at 12 weeks, and persisted through 24 weeks [13]. Although our study did not include PRO measures at time points of <1 year, our findings suggest that the improvement in PROs after rituximab initiation extends through 1 year.

This analysis is among the first to describe patient real-world experience with rituximab treatment 1 year after initiation. Patients in this observational cohort were treated and followed consistent with routine care rather than based on a mandated treatment protocol or visit schedule. Most patients (≈80%) persisted with rituximab through 1 year. However, because the follow-up period for this analysis was limited to 1 year, the long-term effect of rituximab, with or without retreatment, on PROs could not be ascertained.

In conclusion, these results suggest that treatment with rituximab can improve HRQOL in addition to controlling or improving underlying disease in patients with long-standing RA previously treated with TNFis.

## Electronic supplementary material

Fig. S1 (DOCX 46 kb)

Table S1 (DOCX 14 kb)
