# Supplementary material for: Impact of rituximab on patient-reported outcomes in patients with rheumatoid arthritis from the US Corrona Registry
Source: Clin Rheumatol. 2017 Jul 17;36(9):2135–40. doi: 10.1007/s10067-017-3742-2 (PMC5554472; doi:10.1007/s10067-017-3742-2)
Supplement: Supplementary file 1 — (DOCX 46 kb) [file 10067_2017_3742_MOESM1_ESM.docx]

**Supplemental Material**

**Supplemental Fig. 1** Summary of patient selection based on inclusion criteria


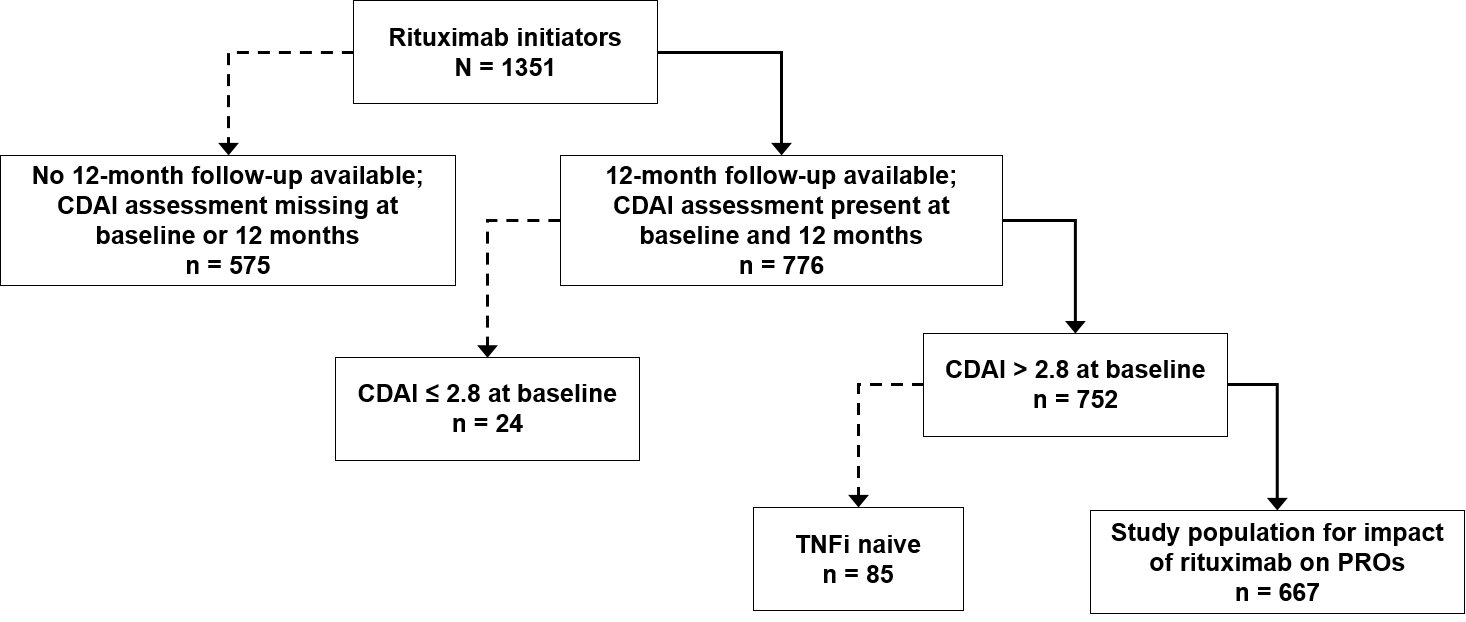


CDAI, Clinical Disease Activity Index; PRO, patient-reported outcome; TNFi, tumor necrosis factor inhibitor.
