# Supplementary material for: Impact of rituximab on patient-reported outcomes in patients with rheumatoid arthritis from the US Corrona Registry
Source: Clin Rheumatol. 2017 Jul 17;36(9):2135–40. doi: 10.1007/s10067-017-3742-2 (PMC5554472; doi:10.1007/s10067-017-3742-2)
Supplement: Supplementary file 2 — (DOCX 14 kb) [file 10067_2017_3742_MOESM2_ESM.docx]

**Supplemental Table 1** Rituximab persistency at 1 year, overall, and by prior TNFi use

|  | **Total**  **N = 667** | **1 Prior TNFi**  **n = 284** | **≥ 2 Prior TNFis**  **n = 383** | ***P* Value^a^** |
| --- | --- | --- | --- | --- |
| Re-treated, n (%)^b^ | 422 (63.3) | 185 (65.1) | 237 (61.9) | 0.078 |
| Not re-treated and did not switch to another biologic, n (%) | 104 (15.6) | 50 (17.6) | 54 (14.1) |  |
| Switched to another biologic, n (%) | 141 (21.1) | 49 (17.3) | 92 (24.0) |  |

TNFi, tumor necrosis factor inhibitor.

^a^ The *P* value represents the comparison between patients who received 1 prior TNFi vs those who received ≥ 2 prior TNFis.

^b^ Includes patients who were re-treated and persisted on rituximab; patients who were re-treated and switched to another biologic were included in the “switched to another biologic” row.
